# In Vitro Anti-Leishmanial Effect of Metallic Meso-Substituted Porphyrin Derivatives against *Leishmania braziliensis and Leishmania panamensis* Promastigotes Properties

**DOI:** 10.3390/molecules25081887

**Published:** 2020-04-19

**Authors:** Fabian Espitia-Almeida, Carlos Díaz-Uribe, William Vallejo, Doris Gómez-Camargo, Arnold R. Romero Bohórquez

**Affiliations:** 1 Grupo de Fotoquímica y Fotobiología, Universidad del Atlántico, Puerto Colombia 810007, Colombia; fespitia@mail.uniatlantico.edu.co (F.E.-A.); carlosdiaz@mail.uniatlantico.edu.co (C.D.-U.); 2 Grupo de Investigación UNIMOL, Universidad de Cartagena, Cartagena 130001, Colombia; dmtropical@unicartagena.edu.co; 3 Grupo de Investigación CODEIM, Universidad Industrial de Santander, Bucaramanga 680002, Colombia; arafrom@uis.edu.co

**Keywords:** porphyrin, metalloporphyrins, photodynamic therapy, *Leishmania braziliensis*, *Leishmania panamensis*, singlet oxygen

## Abstract

In this study, a family of porphyrins based on 5,10,15,20-Tetrakis(4-ethylphenyl)porphyrin (**1**, Ph) and six metallo-derivatives (Zn^2+^(**2**, Ph-Zn), Sn^4+^(**3**, Ph-Sn), Mn^2+^ (**4**, Ph-Mn), Ni^2+^ (**5**, Ph-Ni), Al^3+^ (**6**, Ph-Al), and V^3+^ (**7**, Ph-V)) were tested as photosensitizers for photodynamic therapy against *Leishmania braziliensis* and *panamensis*. The singlet oxygen quantum yield value (Φ_Δ_) for (**1**–**7**) was measured using 1,3-diphenylisobenzofuran (DPBF) as a singlet oxygen trapping agent and 5,10,15,20-(tetraphenyl)-porphyrin (H2TPP) as a reference standard; besides, parasite viability was estimated by the MTT assay. After metal insertion into the porphyrin core, the Φ_Δ_ increased from 0.76–0.90 and cell viability changed considerably. The Φ_Δ_ and metal type changed the cytotoxic activity. Finally, (**2**) showed both the highest Φ_Δ_ (0.90) and the best photodynamic activity against the parasites studied (IC_50_ of 1.2 μM).

## 1. Introduction

*Leishmania spp* are extra and intracellular protozoan parasites that infect a variety of animals (e.g., dogs, rodents, reptiles). However, this zoonosis also affects human beings when they invade the habitat of both natural reservoirs and transmitting vectors thereof [1,2]. The parasite vectors are female hematophagous mosquitoes of the genera *Phlebotomus* and *Lutzomyia* [3,4]. The appearance of this disease in humans can be observed on the skin surface, in mucous membranes, and in some organs (liver and vessels). Among these, the cutaneous is the most frequent form of appearance. Those three clinical presentations have distribution in more than 100 countries in five continents [4], with an estimate of 350 million people at risk of suffering from it. Currently, there are 12 million people infected, with an annual incidence of 2 million people, with around 65,000 deaths reported per year [1,2,5,6]. This disease is considered a priority problem for public health around the world, with greater interest in poorest countries with high levels of malnutrition, economic and social inequality once they face the biggest impacts and incidence [7,8]. Currently, the pharmacological treatments against this disease (e.g., glucantime, miltefosine, pentamidine, isethionate, amphotericin B) have shown some degree of effectiveness against the parasites. However, due to their high toxicity and a wide range of adverse effects, such treatments are controlled and restricted [9,10,11,12,13,14,15,16]. Besides, given the resistance observed in recent years by *Leishmania spp* against all these therapeutic options [17], it is of utmost importance to search for new therapeutic alternatives that are more effective, less toxic, and both safer and more affordable for all vulnerable populations [18]. In this sense, due to their biological and photodynamic properties to produce reactive oxygen species (ROS), especially singlet oxygen when they are irradiated with visible light, porphyrin and metalloporphyrin derivatives have been used as an alternative tool in photodynamic therapy (PDT) against *Leishmania spp* [19,20]. Previous studies reveal that metals affect the stability of the porphyrin macrocycle and, therefore, metals can alter the photophysical properties of the sensitizer; while enhanced intersystem crossing to the triplet state might be expected, followed by metalation of porphyrins due to the heavy-atom effect. Complexes with diamagnetic metals (e.g., Zn) have higher singlet oxygen quantum yields, since diamagnetic metals promote intersystem crossing and have a long triplet lifetime [21,22,23,24]. This behavior is not reported for paramagnetic metals (e.g., Sn, Al, V, Mn). In the case of substituents of intermediate size into the porphyrin core, the triplet lifetimes are observed to decrease by up to two orders of magnitude. This is attributed to a distortion of the macrocycle symmetry when the substituents “squeeze through” upon the hindered rotation of the phenyl group [25,26]. Besides, amphiphilic groups can facilitate better delivery and accumulation of porphyrins in the cells. Several studies have shown that the efficiency in photoactivity increases when the number of carbon atoms in the side chains is increased [27]. The presence of a long alkyl chain was shown to be important for high PDT efficiency of the amphiphilic tripyridyl porphyrins. Lesar et al. showed that lipophilic moiety significantly improved PDT efficiency compared with the hydrophilic analog which lacked the long alkyl chain [28]. Literature data suggest that hydrophilic porphyrins linked to long hydrophobic chains are incorporated much easier into micelle formed by fatty substances. According to expectations, the attachment of alkyl chain to the porphyrin molecule considerably increased its hydrophobic properties [29]. Some reports suggest that when the substituent chain is increased, a greater affinity of the sensitizer to the membranes is allowed, that is why its photodynamic activity increases [30]. Ezzeddine et al. reported a progressive increase of lipophilicity from shorter hydrophilic (methyl) to longer amphiphilic (hexyl) alkyl chains which increased the phototoxicity of the Zn(II) N-alkylpyridylporphyrins [31]. However, the photophysical properties, such as fluorescence lifetime and quantum yield, of singlet oxygen do not change significantly [32,33]. Furthermore, β-substituted porphyrin systems have been evaluated against *L. panamensis* in the amastigote stage, showing IC_50_ values between 5.7 and 24.1 µM [34]. Besides, regarding these compounds, there have been reports of cellular viabilities <10% against *L. major* and *L. braziliensis* in the promastigote stage [35]. Other systems like the benzoporphyrins have shown suitable IC_50_ values (3.35 µM) against *L. major* [36]. Substantial improvements have been reported for these systems when metals are introduced into the macrocycle core. Gomes et al. reported improvement of activity against *L. amazonensis* for inclusion of Bi^3+^ and Sb^5+^, with IC_50_ values of 93.8 µM and 52.4 µM against *L. amazonensis* [37]. Moreover, the cytotoxic activity of these systems against *L. braziliensis* in the promastigote stage was improved after the inclusion of Zn^2+^ [38]. In a recent publication, our group reported in detail the photophysical and DFT results for (**1**–**7**). In that study, we proposed that (**1**–**7**) could be tested as sensitizers for photodynamic therapy [39]. In view of that, in the present study, our aim is to demonstrate the cytotoxic activity of (**1**–**7**) against *L. braziliensis* and *L. panamensis*.

## 2. Results and Discussion

### 2.1. Singlet Oxygen Quantum Yield

The efficient interaction of the photosensitizer triplet state with the molecular oxygen ground state may result in generation of singlet oxygen [40]. In order to determine Φ_Δ_, DPBF was used as a singlet oxygen trapping agent and H2TPP as a reference standard. The generation of singlet oxygen by (**1**–**7**) is evidenced by chemical trapping of singlet oxygen by DPBF, and the Φ_Δ_ values of the compounds are listed in Table 1. The results indicate that (Ph-Zn, Ph-Mn, Ph-Al, Ph-V) had a quantum yield higher than (**1**, Ph). This difference could be related to an increase of relaxation of excited states in macromolecule; moreover, the insertion of these metals inside the ring generated more stability for the generation of singlet oxygen [25,41,42].

In general, the Φ_Δ_ of (**1**–**7**) were lower for the paramagnetic metals than for the diamagnetic ones, and this is in line with previous studies, which showed that porphyrins containing paramagnetic ions were very poor photosensitizers [24,26]. It is possible that the introduction of low energy charge-transfer states associated with disruption of the planarity of the macrocyclic ring system provides alternative non-radiative deactivation channels. Finally, since Φ_Δ_ values as low as 0.11 are known for porphyrins derivatives in clinical trials, such as Lutetium Texaphyrin [43], and because singlet oxygen has been implicated as an intermediary species leading to cell death following photoexcitation sensitizers agents in photodynamic therapy [24], the results shown in Table 1 indicate that (**1**–**7**) are suitable as potential materials for photodynamic therapy.

### 2.2. Antileishmanicidal Activity

**Figure 1:**
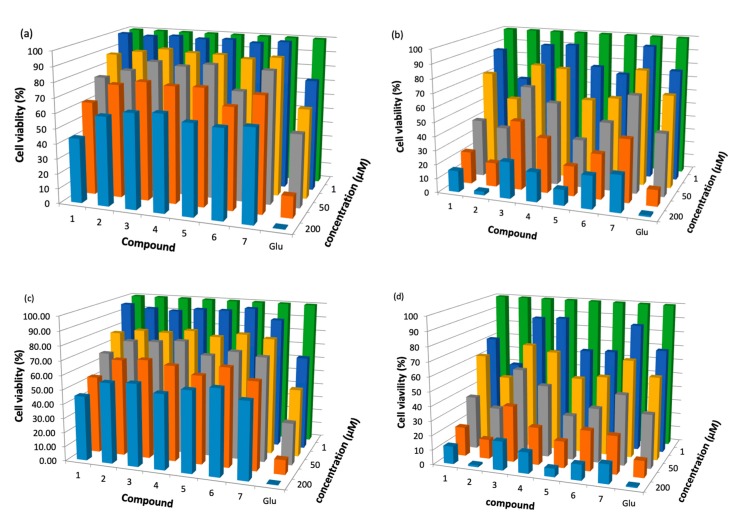
Parasite viability percentage results for compounds (**1**–**7**) with incubation periods of 24 h: against *L. braziliensis* (**a**) in the dark, (**b**) under light irradiation; against *L. panamensis* (**c**) in the dark; (**d**) under light irradiation.

**Figure 2:**
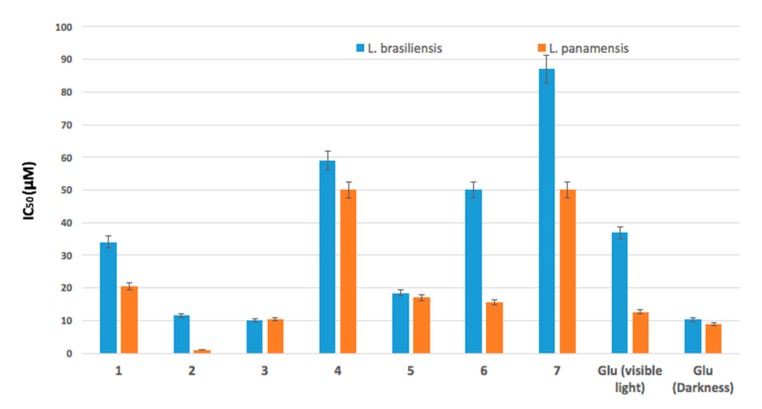
IC_50_ values for photoinactivation of L. braziliensis and *L. panamensis* promastigotes after 24 h of incubation in the presence of (**1**–**7**) and the positive control under light irradiation.

Several compounds have already used sensitizers against *Leshmania* species [44,45]. However, the search for new substances is an important topic in this research field. Compounds (**1**–**7**) were studied in the promastigotes stage of *L. panamensis* and *L. braziliensis*, with viability assessed by the MTT assay. Figure 1 shows in detail the viability (%) results of *L. braziliensis* and *L. panamensis* with incubation periods of 24 h in the presence of (**1**–**7**) both in the dark and under visible irradiation. The results show that (**1**–**7**) had the ability to effectively inhibit the parasites. In addition, a decrease in the viability of the parasites was observed with the increase in the concentrations of the treatment. Figure 1a,c show that, under light irradiation, the viability of (**1**–**7**) was similar to the viability of the Glucantime control for all ranges of concentration. These results are relevant, it verifies the potential of the (**1**–**7**) as sensitizers for PDT. Furthermore, the inhibitory activity was lower without light irradiation for (**1**–**7**), and this is due to the interaction of light with endogenous biomolecules [46]. When 200 μM of the compounds were used, (**2**, Ph-Zn) had the highest inhibitory activity against both *L. braziliensis* and *L. panamensis,* even the cell viability of (**2**, Ph-Zn) was the same as that of the Glucantime control. According to Table 1, (Ph-Zn) had the highest Φ_Δ_ (0.90), then under visible irradiation, the amount of singlet oxygen available to attack the *leishmania* parasite is larger and the cytotoxic effect could be bigger. The IC_50_ value (concentration that inhibited cell growth by 50%) was determined, and the results are shown in Figure 2. In all cases of the tests, the activation of sensitizers by irradiation ensures lower IC_50_ values. In the absence of light, the cytotoxic activity against the parasite was lower; the IC_50_ for (**1**–**7**) was higher than 200 μM in the dark; all compounds required light activation—these results are in line with other reports [20,47]. Compounds (**1**–**7**) showed high toxicity against the parasites under light irradiation, and (**1**, **3**–**7**) had IC_50_ similar of larger than the positive control against both parasites; only (Ph-Zn) had lower IC_50_ (1.2 μM) comparing to the positive control under irradiation (12.7 μM) and in the dark (8.0 μM) against *L. panamensis*. This result is associated with the biggest Φ_Δ_ value of (**2**). Table 2 lists the IC_50_ values for compounds (**1**–**7**) under visible irradiation and without irradiation (in the dark).

Besides, Figure 2 indicates that (**1**–**7**) were more effective against *L. panamensis* than against *L. braziliensis*. This result could be associated with the multi-resistance mechanism reported for *L. braziliensis* [48,49,50,51]. The parasite inhibition mechanism is unknown and there is no clear report in the literature [52]. However, after compounds (**1**–**7**) were irradiated with visible light (see Table 1), singlet oxygen was generated—this oxidant species could generate substantial damage to parasites at the cellular membrane level and even irreparable damage to vital proteins or DNA that induce death [52,53,54]. Our results suggest that singlet oxygen could be a reason for inactivation of the parasite. It is clear that those compounds operate efficiently under visible light; in the dark the damage to the parasites was not comparable to that of the positive control. Finally, these results are relevant and show the potential of (**1**–**7**) as sensitizers for PDT, which indicate that (Ph-Zn) is the best candidate for PDT applications.

## 3. Materials and Methods

### 3.1. Synthesis

**Scheme 1:**
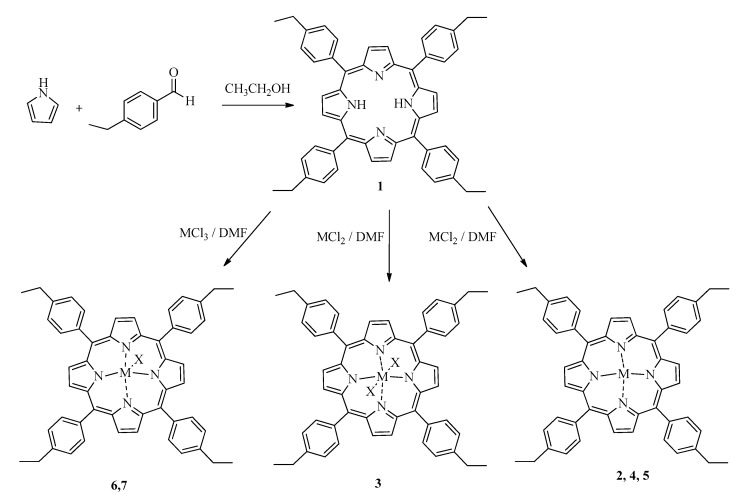
Chemical synthesis of: **(1**) 5,10,15,20-Tetrakis(4-ethylphenyl)porphyrin and metal-derivatives, (**2**) Zn (II), (**3**) Sn (IV), (**4**) Mn (III), (**5**) Ni (II), (**6**) Al (III), (**7**) V (III). Into scheme, X means Cl^−^.

All reagents were supplied by Aldrich. We synthesized porphyrin according to Alder and Cols method [55], which relies on stirring aldehyde and pyrrole in propionic acid for 6 h at room temperature and an oxygen atmosphere (see Scheme 1) [39]:

*5,10,15,20-tetrakis(4-ethylphenyl)porphyrin* (**1**): A mixture of pyrrole (8 mmol) and 4-ethylbenzaldehyde (8 mmol) in of propionic acid (60 mL) was stirred by 6 h at room temperature in an open container. The product was extracted from the reaction medium after addition of methanol (40 mL). We obtained 0.820 g of a bright purple powder that was purified through column chromatography using silica gel (2.5 × 24 cm) as stationary phase, and petroleum ether:ethyl acetate 5:1 (rf = 0.66). Yield: 0.680 g, 46%; melting point > 300 °C; UV-Vis (ethyl acetate) λ (nm): 415, 512, 547, 590, 646; FT-IR-ATR (cm^−1^): N-H (3312.97), C*_sp3_*-H (2960.44), C=C (1685.54), C=N (1180.23), C-N (1020.47); ^1^ H RMN (400 MHz, CDCl_3_) δ (ppm): 1.57 (12 H, t, *J* = 7.6 Hz, -CH_2_CH_3_), 3.02 (8 H, q, *J* = 7.6 Hz, -CH_2_CH_3_), 7.60 (8 H, d, *J* = 7.9 Hz, 3-H_Ar_), 8.16 (8 H, d, *J* = 7.9 Hz, 4-H_Ar_), 8.90 (8 H, s, Py); ^13^ C RMN (100 MHz, CDCl_3_) δ(ppm): 15.56 (-CH_2_CH_3_ × 4), 28.96 (-CH_2_CH_3_ × 4), 120.21 (2-C_Ar_ × 8), 126.23 (1-C_Ar_ × 4), 131.28 (C_β_-Py × 8), 134.59 (3-C_Ar_ × 8), 139.63 (C_α_-Py × 8), 143.62 (4-C_Ar_ × 4); MS (ESI-IT), *m*/*z*: 727.2 [M + H]^+^; Anal. Elem. Calc. for C_52_H_46_N_4_ (%): C (85.91), H (6.39), N (7.71), Anal. Elem. Found. (%) C_52_H_46_N_4,_ C (85.95), H (6.34), N (7.71).

Compound (**2**–**7**) were synthesized by mixing (**1**) with the metal chloride salt for each metal in DMF. The mixture was stirred for 6 h at room temperature. Then, the reaction mixture was cooled in ice-water bath; the formed precipitate was filtered and dried at room temperature; (**2**–**7**) were purified through column chromatography with silica gel (2.5 × 24 cm), petroleum ether:ethyl acetate (PE:EA) was used as mobile phase. Details of the spectroscopic characterization are listed in supplementary materials.

### 3.2. Singlet Oxygen Quantum Yield

The Φ_Δ_ values of (**1**–**7**) were determined in air using the relative method with 1,3- diphenylisobenzofuran (DPBF) as a singlet oxygen trapping agent and 5,10,15,20-(tetraphenyl)-porphyrin (H2TPP) as a reference standard. The tests consisted of preparing a 1 × 10^−9^ M solution of each compound in Dimethylformamide (DMF) by triplicate, and calculations were determined according to Equation (1) [56,57,58,59]:

(1) $$\Phi \Delta ={\;\Phi }_{\Delta \mathrm{st}}\times \frac{W}{W_{\mathrm{st}}\;}$$

where Φ_∆st_ is the singlet oxygen quantum yield of standard H_2_TPP in DMF (0.64), W y W_st_ are the DPBF photobleaching rates in the presence of complex (1 and 2) and standard porphyrin, respectively. Data for *Singlet Oxygen Quantum Yield calculation* are provided in supplementary materials.

### 3.3. Fluorescence Quantum Yield

The comparative method was used to determine fluorescence quantum yield. Fluorescein dissolved in water was standard, and sensitizers were dissolved in ethyl acetate. The fluorescence quantum yield values were determined by taking the maximum of the Soret band as the excitation wavelength (range 420–750 nm; slit = 2 nm). Quantum fluorescence yield was calculated with the following equation [37,42,60,61]:

(2) $$\Phi x\;={\;\Phi f}_{\mathrm{est}}*\frac{F_{x}\;{*\;A}_{\mathrm{est}}*\;{ɳ}_{\;\mathrm{est}}^{2}}{F_{\mathrm{est}}\;{*\;A}_{x}\;*\;{ɳ}_{\;x}^{2}}\;\left(\mathrm{ec}.\;1\right)$$

where F_x_ and F_est_ correspond to the area under the curve in the fluorescence emission spectrum for compounds (**1**), (**2**) and standard. A_x_ and A_est_ correspond to absorbance at excitation wavelength for compounds (**1**), (**2**) and standard; ɳ_x_ and ɳ_est_ correspond to the refraction index for solvents (ɳ*_ethyl acetate_* = 1.3724 and ɳ*_water_* = 1.33336). Data for *Singlet Oxygen Quantum Yield calculation* are provided in supplementary materials.

### 3.4. Parasites

*Leishmania panamensis* (M2903) and *Leishmania braziliensis* (UA140) were used in the in vitro study. The parasites were cultured in RPMI-1640 supplemented with 10% fetal bovine serum, 1% glutamine and 4% antibiotics (200 U penicillin/200 μg Amikacin) under incubation conditions of 5% CO_2_. The metacyclic promastigotes in the infectious stage were isolated from stationary cultures of 5 days using a uniform procedure based on a modified density gradient purification.

### 3.5. Parasite Viability

Parasite viability was estimated by the MTT assay, converting a yellow tetrazolium salt, 3-(4,5-dimethylthiazol-2-yl)-2,5-diphenyltetrazolium bromide into an insoluble product (formazan); the amount of formed formazan depends on the number of viable parasites present [58,59,62]. The antileishmanicidal activity was studied at different concentrations in the presence and absence of light. The irradiation source was Omnilux lamps (EL10000AG), with a range of λ_emission lamp_ = 420 nm–450 nm for using light intensity 80 J·cm^−2^. All the measurements of the optical densities were taken in microplates of 96 U-bottom wells, using the Multiskan Sky ThermoScientific equipment. Standard deviation was obtained from 12 independent experiments—these were correlated with a percentage variation coefficient <5%. We applied an ANOVA test to determine the differences or similarities between treatments and the positive control. In addition, a post hoc analysis was performed using Tukey statistics. Finally, differences were considered to be significant when *p* < 0.05.

## 4. Conclusions

Porphyrins (**1**–**7**) showed suitable singlet oxygen quantum yields, which induced inhibition of the *L. braziliensis* and *L. panamensis* growth when the compounds were irradiated with a visible light source. The non-irradiated treatments generated little or no inhibitory response of the parasites. All the results indicate that (**1**–**7**) have suitable properties to be used in photodynamic therapy. All the compounds showed better cytotoxic against *L. panamensis* than against *L. braziliensis*. Compound (**2**) was the best photosensitizer of all the compounds included in this study, as it showed a larger Φ_Δ_ value (0.90) and a better IC_50_ value compared to that of the positive control. Therefore, compound (**2**) is the best candidate to be tested in photodynamic application against *L. braziliensis*.

## Figures and Tables

**Table 1:** Singlet oxygen quantum yield (Φ_Δ_) and fluorescence quantum yield (Φ_f_) for (**1**–**7**).

Compound	ε (M^−1^cm^−1^ × 10^4^)	Φ_f_	Φ_Δ_
**1**, Ph	5.0	0.11 ± 0.02	0.76 ± 0.09
**2**, Ph-Zn	2.5	0.11 ± 0.03	0.90 ± 0.03
**3**, Ph-Sn	6.0	0.32 ± 0.02	0.76 ± 0.05
**4**, Ph-Mn	4.0	0.17 ± 0.02	0.83 ± 0.03
**5**, Ph-Ni	7.0	0.08 ± 0.02	0.68 ± 0.02
**6**, Ph-Al	3.4	0.010 ± 0.005	0.84 ± 0.04
**7**, Ph-V	3.1	0.0020 ± 0.0005	0.86 ± 0.01

**Table 2:** IC_50_ values for photoinactivation of *L. braziliensis* and *L. panamensis* promastigotes in the dark and under visible irradiation.

Compound	*L. braziliensis*		*L. panamensis*	
	IC_50_ (μM) Under Irradiation	IC_50_ (μM) in the Dark	IC_50_ (μM) under Irradiation	IC_50_ (μM) in the Dark
**1**, Ph	34.1 ± 1.8	117	20.6 ± 1.3	105
**2**, Ph-Zn	11.6 ± 1.0	>200	1.2 ± 0.2	>200
**3**, Ph-Sn	10.1 ± 0.7	>200	10.4 ± 0.8	>200
**4**, Ph-Mn	59.0 ± 2.5	>200	50.0 ± 1.3	>200
**5**, Ph-Ni	18.4 ± 2.5	>200	17.0 ± 1.0	>200
**6**, Ph-Al	50 ± 1.2	>200	15.6 ± 0.9	>200
**7**, Ph-V	87 ± 3.5	>200	50.0 ± 2.1	>200
Glucantime	--	10.3 ± 0.9	--	8.8 ± 0.8

## Supplementary Materials

The following materials are available online, FTIR, Florescence, UV-Vis, singlet oxygen plots data and the synthesis details.
