# Supplementary material for: In Vitro Anti-Leishmanial Effect of Metallic Meso-Substituted Porphyrin Derivatives against Leishmania braziliensis and Leishmania panamensis Promastigotes Properties
Source: Molecules. 2020 Apr 19;25(8):1887. doi: 10.3390/molecules25081887 (PMC7221524; doi:10.3390/molecules25081887)
Supplement: Supplementary file 1 [file molecules-25-01887-s001.pdf]

Supplementary material

***In vitro* anti-leishmanial effect of metallic meso-substituted porphyrin derivatives against *Leishmania braziliensis* and *Leishmania panamensis* promastigotes properties**

Synthesis

5,10,15,20-tetrakis(4-ethylphenyl)porphyrin-Zn(II) ***(2)***: A mixture of **(1)** (0.2755 mmol) and $ZnCl_2$ (1.4673 mmol) in DMF (20 mL) was stirring by 4 hours at room temperature. The reaction mixture was cooled in ice-water bath; the formed precipitate was filtered and dried at ambient temperature; **(2)** was purified through column chromatography with silica gel (2.5 x 24 cm), petroleum ether:ethyl acetate (PE:EA) was using as mobile phase 3:1 (rf = 0.87). Yield: 0.150 g, 75 %; melting point > 300 ºC; UV-Vis (ethyl acetate) λ (nm): 422, 556, 597; FT-IR-ATR (cm^{-1}): C_{sp3}-H (2960.44), C=C (1650.32), C=N (992.39), C-N (851.54); Anal. Elem. Calc. for $C_{52}H_{44}N_4Zn$ (%): C (79.05), H (5.61), N (7.10), Zn (8.27); Anal. Elem. Found $C_{52}H_{44}N_4Zn$ (%): (79.02), H (5.60), N (7.09), Zn (8.28).

5,10,15,20-tetrakis(4-ethylphenyl)porphyrin-Sn(IV) **(3)*:*** A mixture of **(1)** (0.2755 mmol) and $SnCl_2.2H_2O$ (0.8865 mmol) in DMF (20 mL) was stirring by 6 hours at room temperature. The reaction mixture was cooled in ice-water bath; the formed precipitate was filtered and dried at ambient temperature; **(3)** was purified through column chromatography with silica gel, PE:EA was used as mobile phase 3:1 (rf = 0.80). Yield: 0.120 g, 60 %; melting point > 300 ºC; UV-Vis (ethyl acetate) λ (nm): 415, 514, 549; FT-IR-ATR (cm^{-1}): C_{sp3}-H (2960.44), C=C (1470.55), C=N (956.25), C-N (846.32); Anal. Elem. Calc. for $C_{52}H_{44}N_4Cl_2Sn$ (%): C (68.29), H (4.84), N (6.13), Cl(7.75), Sn (12.97).

5,10,15,20-tetrakis(4-ethylphenyl)porphyrin-Mn(II) **(4):** A mixture of **(1)** (0.2755 mmol) and $MnCl_2.4H_2O$ (1.0105 mmol) in DMF (20 mL) was stirring by 6 hours at room temperature. The reaction mixture was cooled in ice-water bath; the formed precipitate was filtered and dried at ambient temperature; **(4)** was purified through column chromatography with silica gel, PE:EA was used as mobile phase 2:1 (rf = 0.89). Yield: 0.118 g, 59 %; melting point > 300 ºC; UV-Vis (ethyl acetate) λ (nm): 416, 469, 549; FT-IR-ATR (cm^{-1}): C_{sp3}-H (2960.44), C=C (1181.04), C=N (964.71), C-N

(846.18); Anal. Elem. Calc. for $C_{52}H_{44}N_4Mn$ (%): C (80.11), H (5.65), N (7.19), Mn (7.05). Anal. Elem. Found. for $C_{52}H_{44}N_4Mn$ (%): C (80.08), H (5.69), N (7.18), Mn (7.04).

5,10,15,20-tetrakis(4-ethylphenyl)porphyrin-Ni(II) **(5)**: A mixture of **(1)** (0.2755 mmol) and $NiCl_2.6H_2O$ (0.8414 mmol) in DMF (20 mL) was stirring by 6 hours at room temperature. The reaction mixture was cooled in ice-water bath; the formed precipitate was filtered and dried at ambient temperature; **(5)** was purified through column chromatography with silica gel, PE:EA was used as mobile phase 3:1 (rf = 0.69). Yield: 0.121 g, 60.5 %; melting point > 300 °C; UV-Vis ethyl acetate) λ (nm): 418, 592; FT-IR-ATR (cm^{-1}): C_{sp3}-H (2960.44), C=C (1454.55), C=N (1002.06), C-N (818.64); Anal. Elem. Calc. for $C_{52}H_{44}N_4Ni$ (%): C (79.72), H (5.62), N (7.16), Ni (7.50). Anal. Elem. Found. for $C_{52}H_{44}N_4Ni$, (%): C (79.70), H (5.66), N (7.15), Ni (7.49).

5,10,15,20-tetrakis(4-ethylphenyl)porphyrin-Al(III) **(6)**: A mixture of **(1)** (0.2755 mmol) and $AlCl_3$ (1.4999 mmol) in DMF (30 mL) was stirring by 8 hours at room temperature. The reaction mixture was cooled in ice-water bath; the formed precipitate was filtered and dried at ambient temperature; **(6)** was purified through column chromatography with silica gel, PE:EA was used as mobile phase 3:1 (rf = 0.79). Yield: 0.141 g, 70.5 %; melting point > 300 °C; UV-Vis (ethyl acetate) λ (nm): 416, 513, 645; FT-IR-ATR (cm^{-1}): C_{sp3}-H (2967.96), C=C (1601.84), C=N (1183.38), C-N (826.31); Anal. Elem. Calc. for $C_{52}H_{44}N_4AlCl$ (%): C (79.34), H (5.60), N (7.12), Al (3.43), Cl (5.51). Anal. Elem. Calc. for $C_{52}H_{44}N_4AlCl$ (%): C (79.32), H (5.63), N (7.12), Al (3.43), Cl (5.50).

5,10,15,20-tetrakis(4–ethylphenyl)porphyrin-V(III) **(7)**: A mixture **(1)** (0.2755 mmol) and VCl_3 (1.2714 mmol) in DMF (30 mL) was stirring by 8 hours at 100 °C. The reaction mixture was cooled in ice-water bath; the formed precipitate was filtered and dried at ambient temperature; **(7)** was purified through column chromatography with silica gel, PE:EA was used as mobile phase 1:3 (rf = 0.79). Yield: 0.138 g, 69 %; melting point > 300 °C; UV-Vis (ethyl acetate) λ (nm): 415, 515, 550, 594; FT-IR-ATR (cm^{-1}): C_{sp3}-H (2959.83), C=C (1603.72), C=N (1184.93), C-N (824.22); Anal. Elem. Calc. for $C_{52}H_{44}N_4VCl$ (%): C (77.0), H (5.43), N (6.91), V (6.29), Cl (4.37). Anal. Elem. Calc. for $C_{52}H_{44}N_4VCl$ (%): C (76.98), H (5.47), N (6.90), V (6.28), Cl (4.37).

Characterization

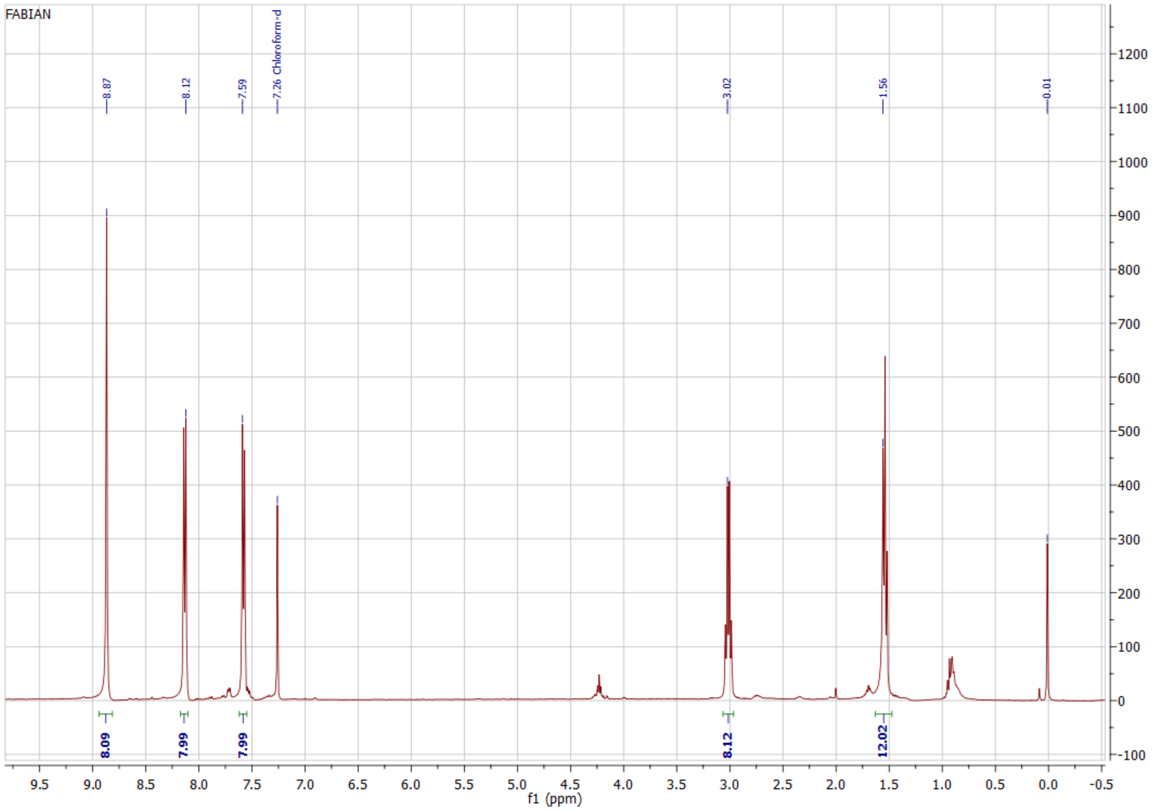

Figure S1. ^{1}H RMN of (1, Ph).

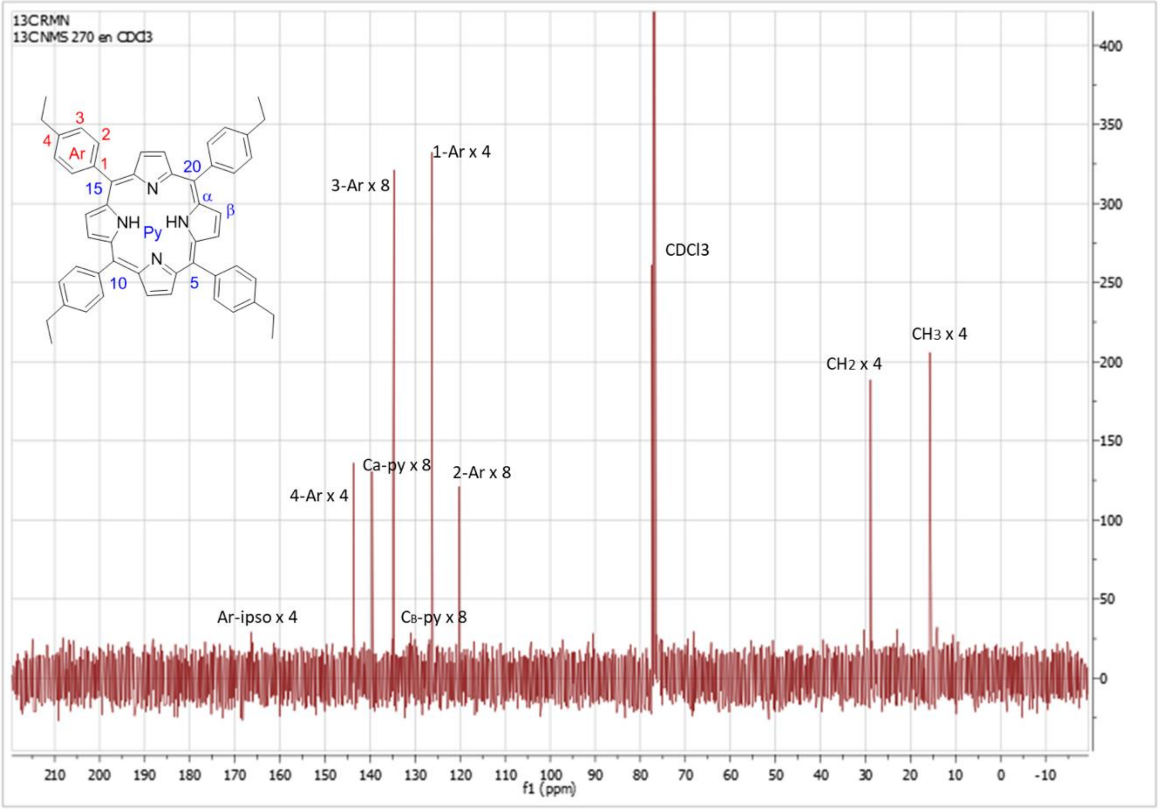

Figure S2. ^{13}C RMN of (1, Ph).

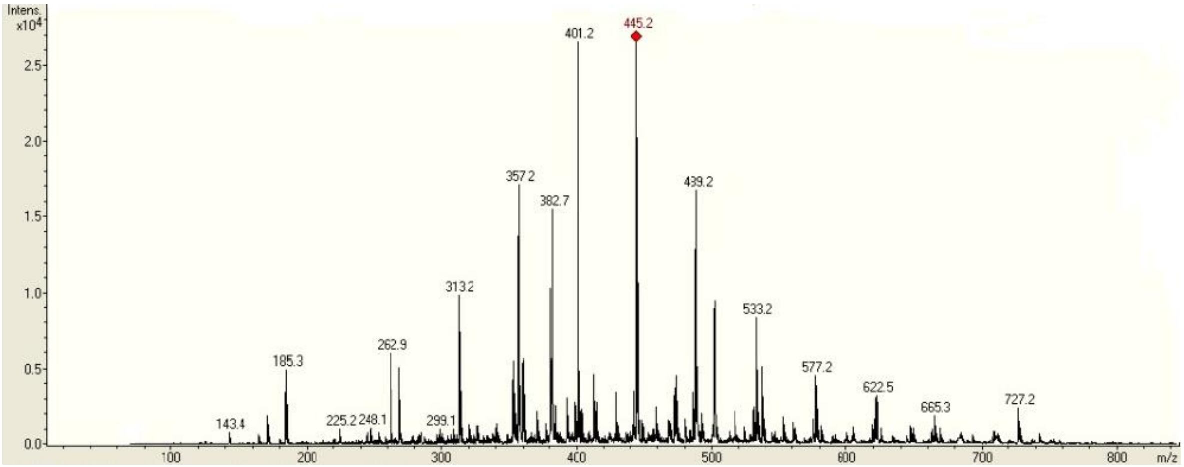

Figure S3. Electrospray ionization tandem mass spectrum of (1, Ph).

FTIR spectra

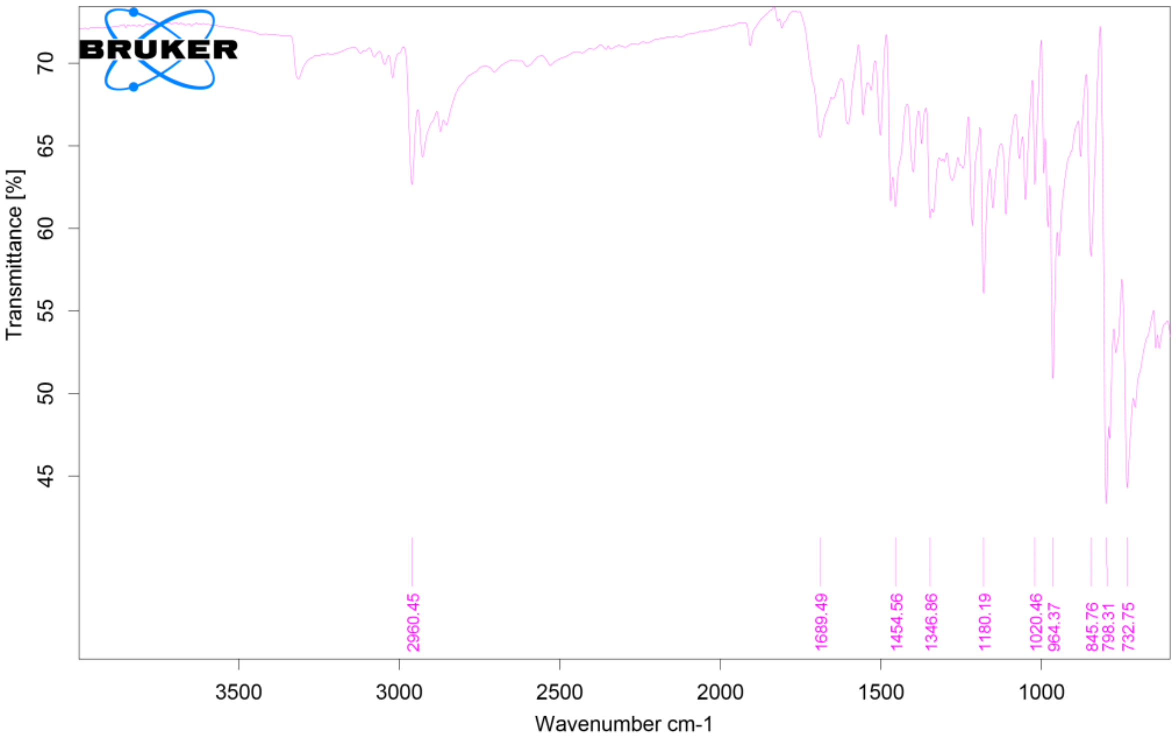

Figure S4. FTIR spectrum of (1, Ph).

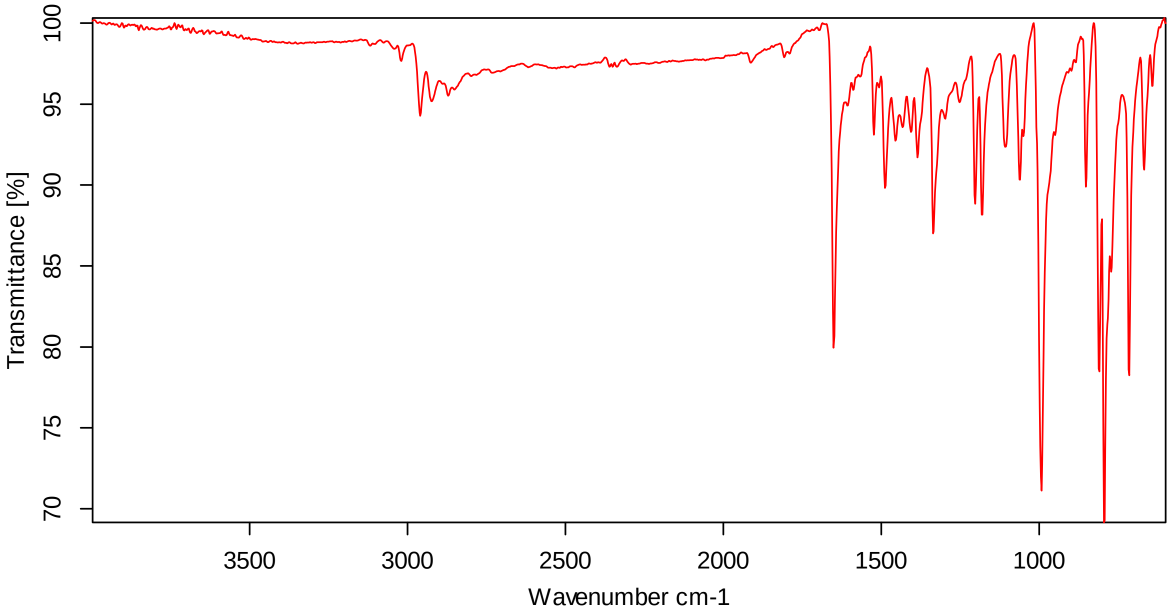

Figure S5. FTIR spectrum of (2, Ph-Zn).

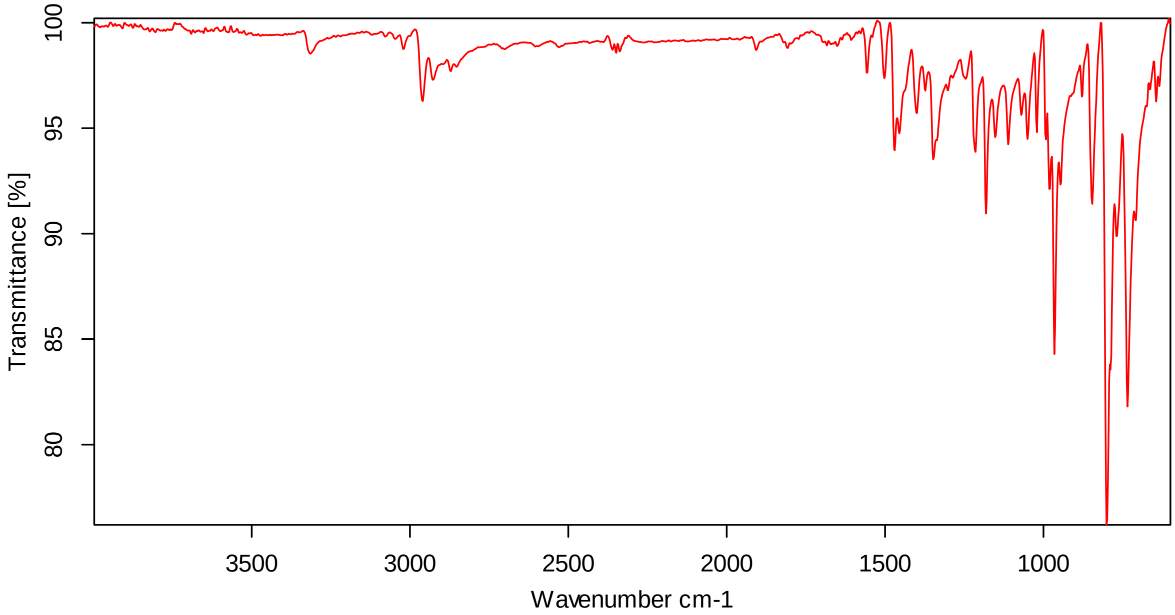

Figure S6. FTIR spectrum of (3, Ph-Sn).

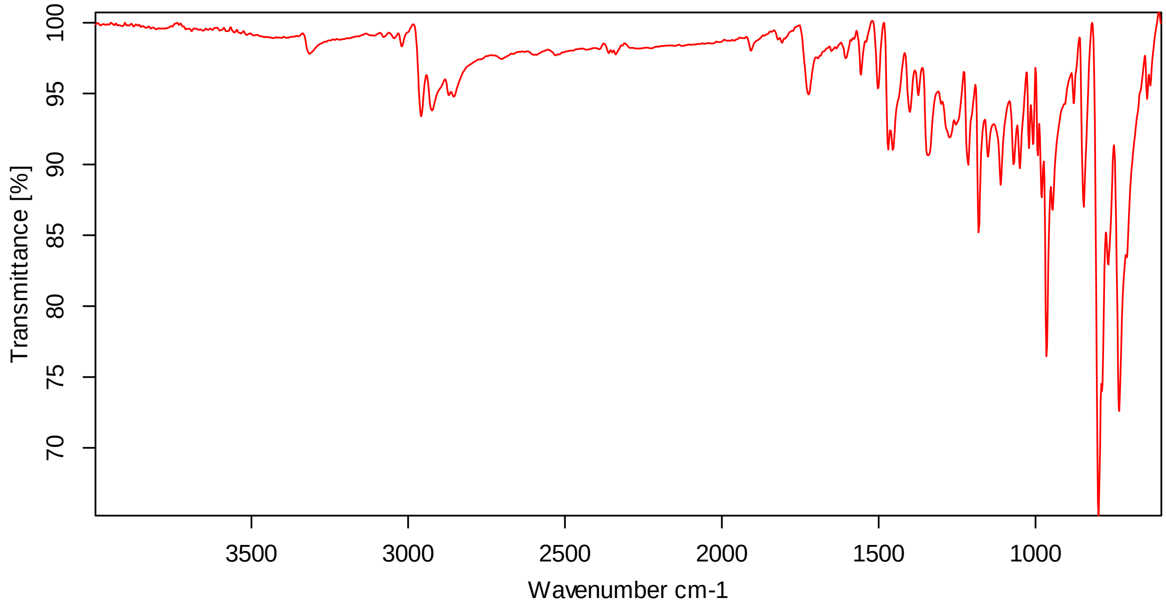

Figure S7. FTIR spectrum of (4, Ph-Mn).

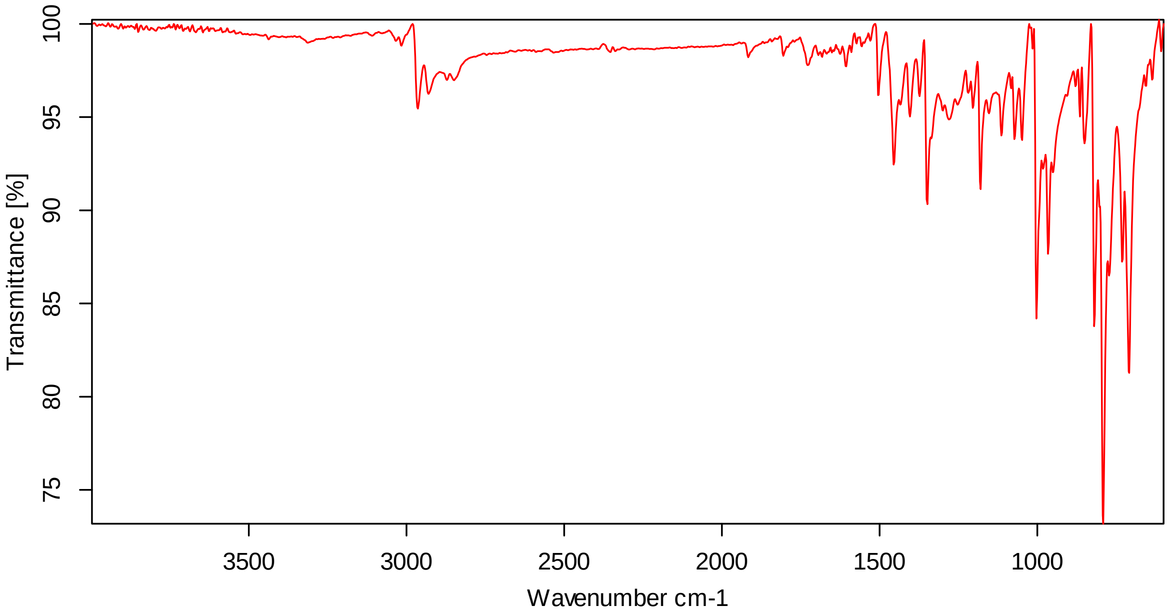

Figure S8. FTIR spectrum of (5, Ph-Ni).

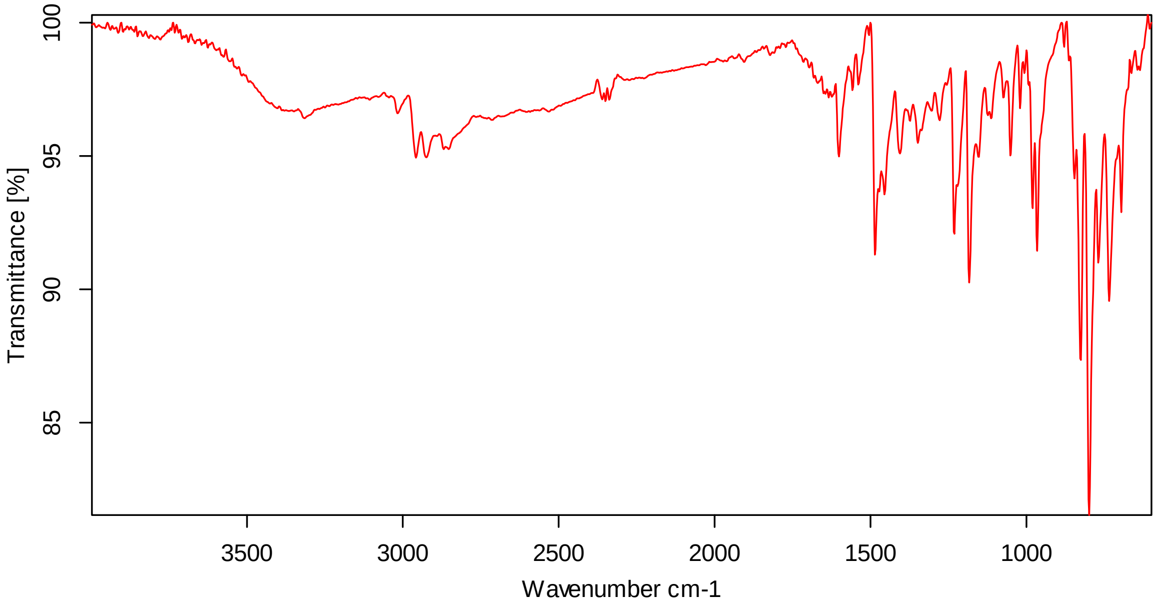

Figure S9. FTIR spectrum of (6, Ph-Al).

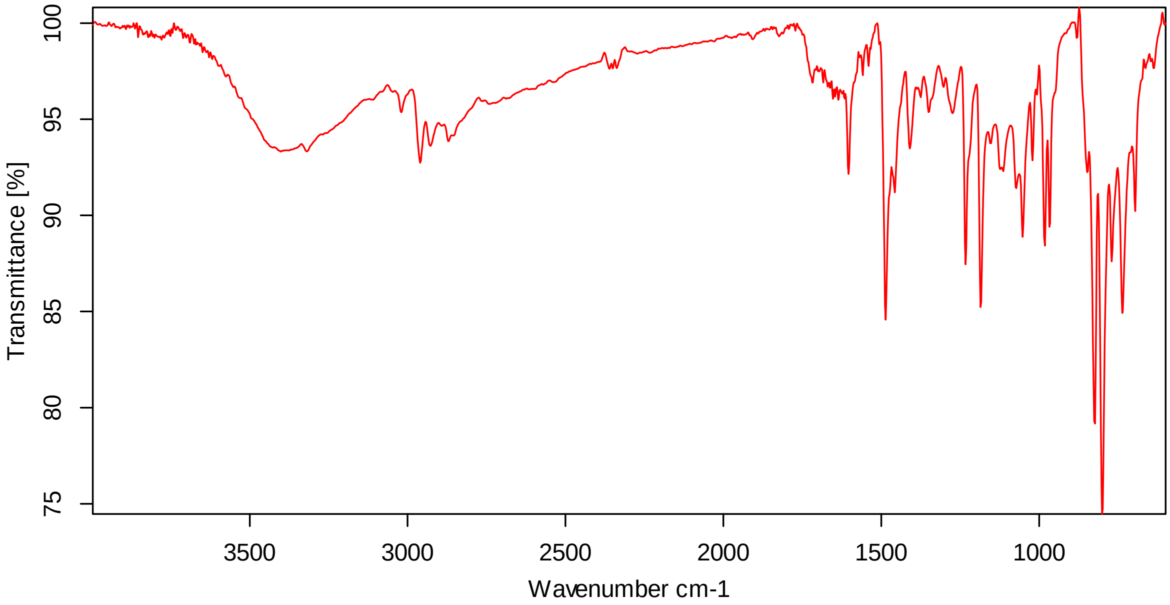

Figure S10. FTIR spectrum of (7, Ph-V).

UV- Vis spectra

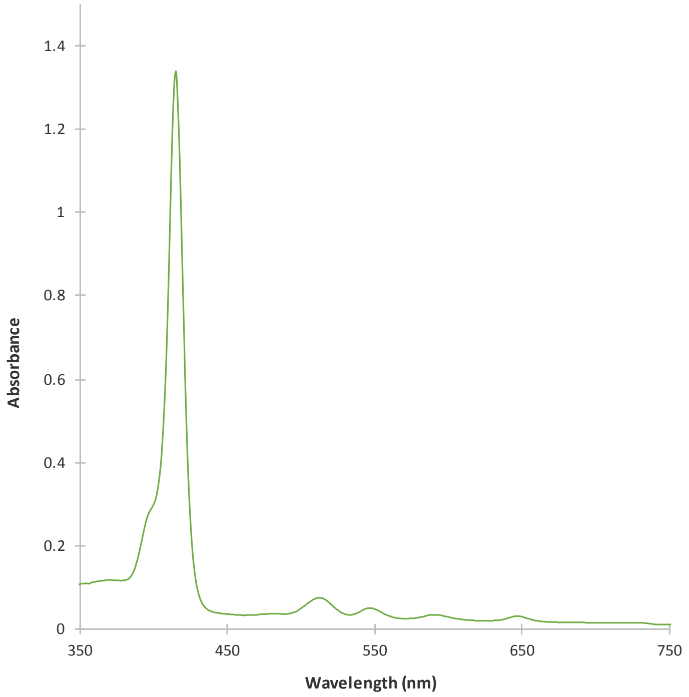

Figure S11. UV-Vis spectrum of (1, Ph) in ethyl acetate.

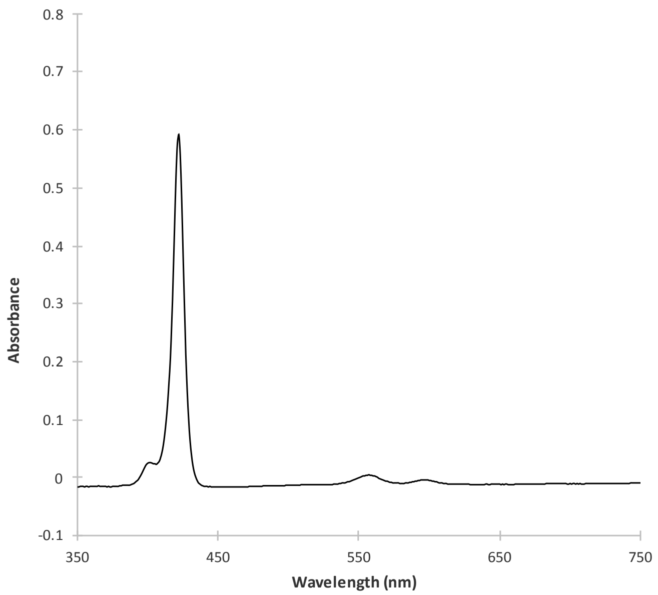

Figure S12. UV-Vis spectrum of (2, Ph-2) in ethyl acetate.

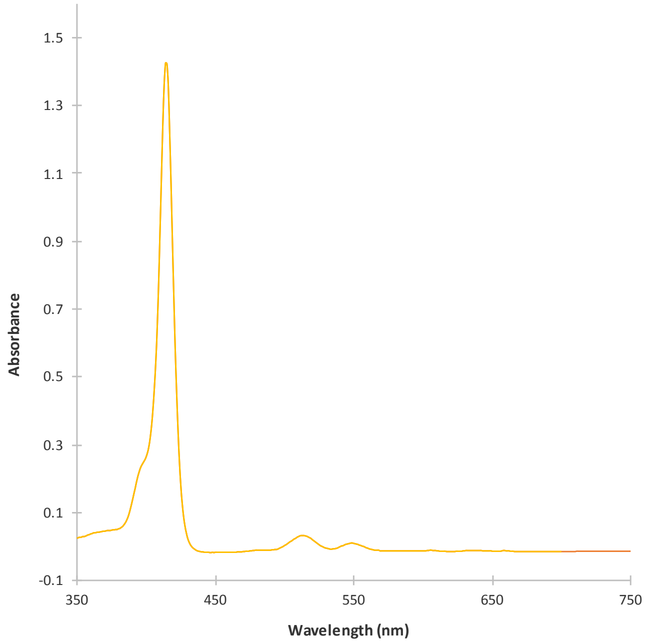

Figure S13. UV-Vis spectrum of (3, Ph-Sn) in ethyl acetate.

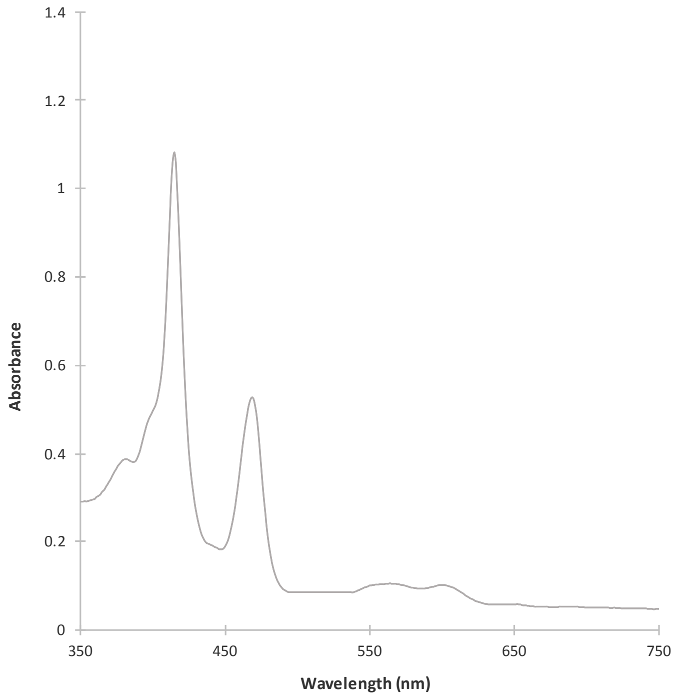

Figure S14. UV-Vis spectrum of (4, Ph-Mn) in ethyl acetate.

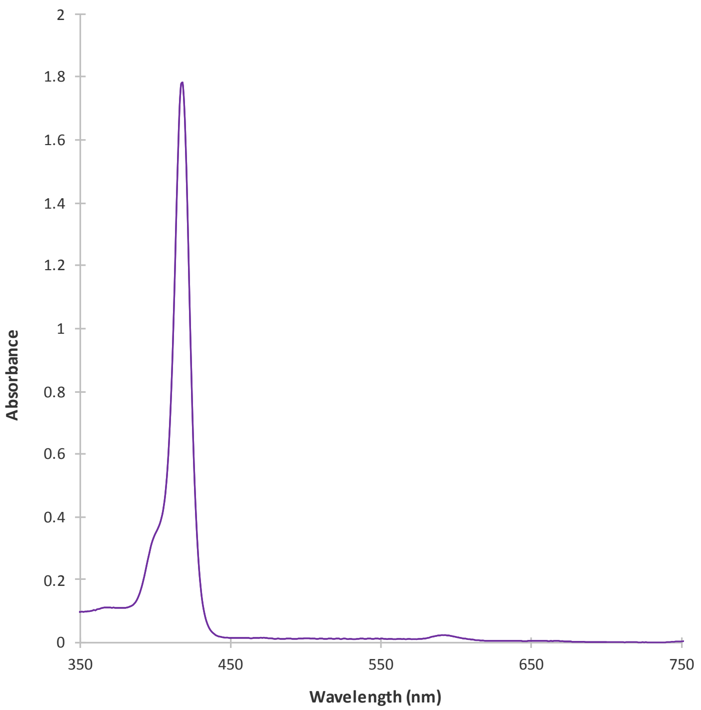

Figure S15. UV-Vis spectrum of (5, Ph-Ni) in ethyl acetate at room pH 7.0.

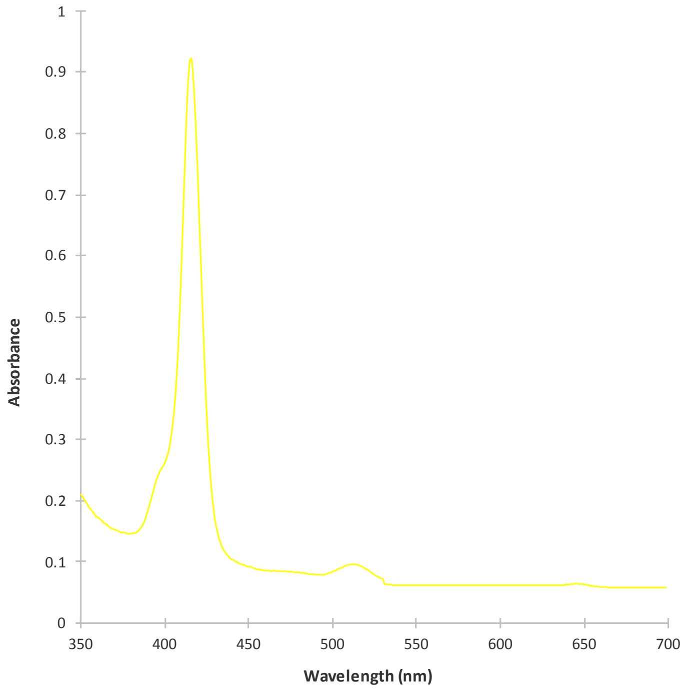

Figure S16. UV-Vis spectrum of (6, Ph-Al) in ethyl acetate.

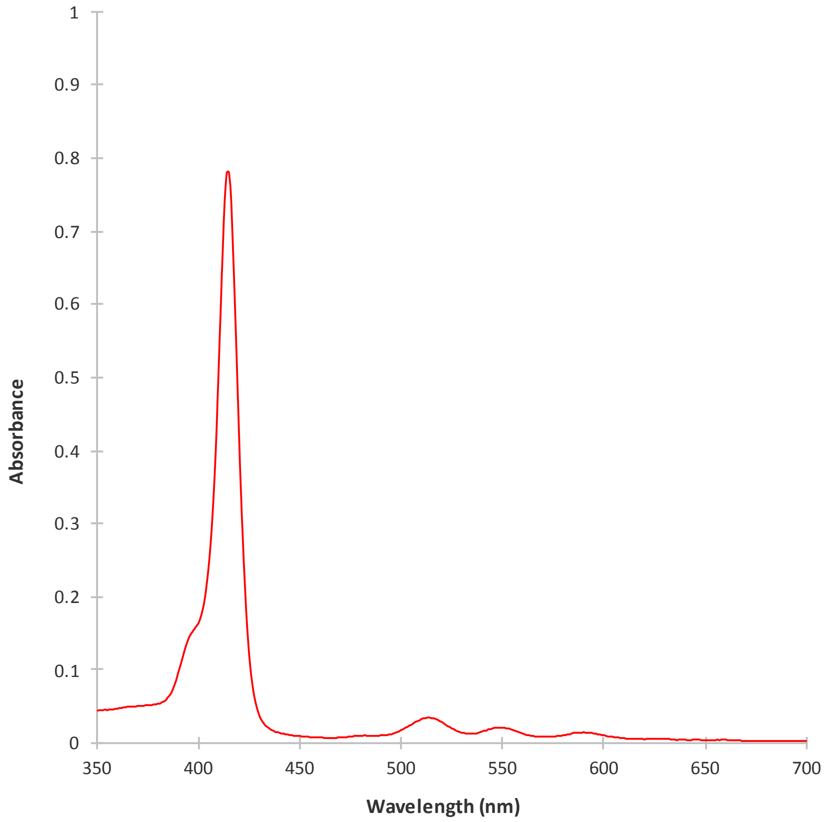

Figure S17. UV-Vis spectrum of (7, Ph-V) in ethyl acetate.

Fluorescence spectra

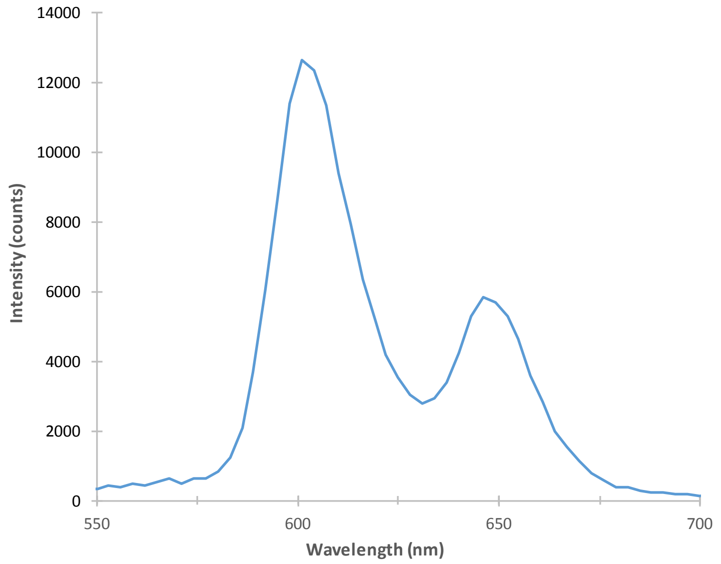

Figure S18. Emission spectrum of (1, Ph) in ethyl acetate.

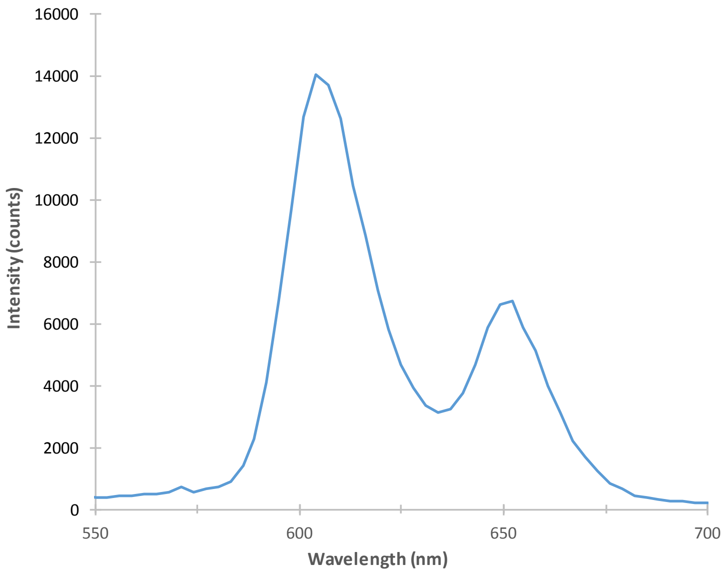

Figure S19. Emission spectrum of (2, Ph-Zn) in ethyl acetate.

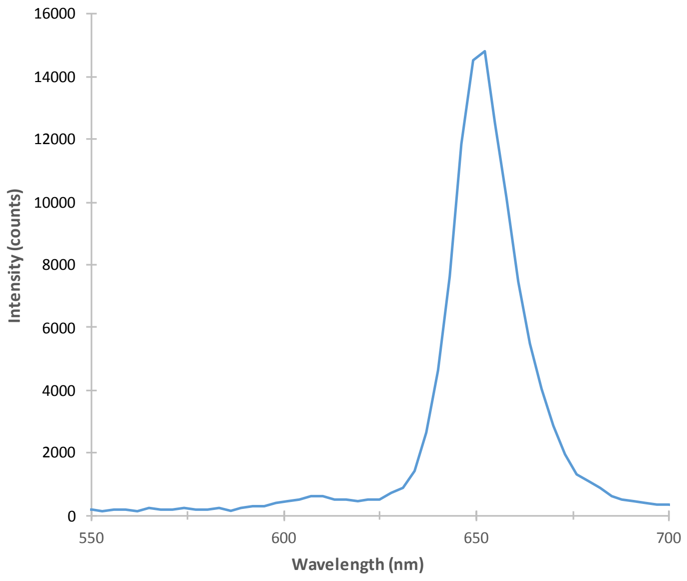

Figure S20. Emission spectrum of (3, Ph-Sn) in ethyl acetate.

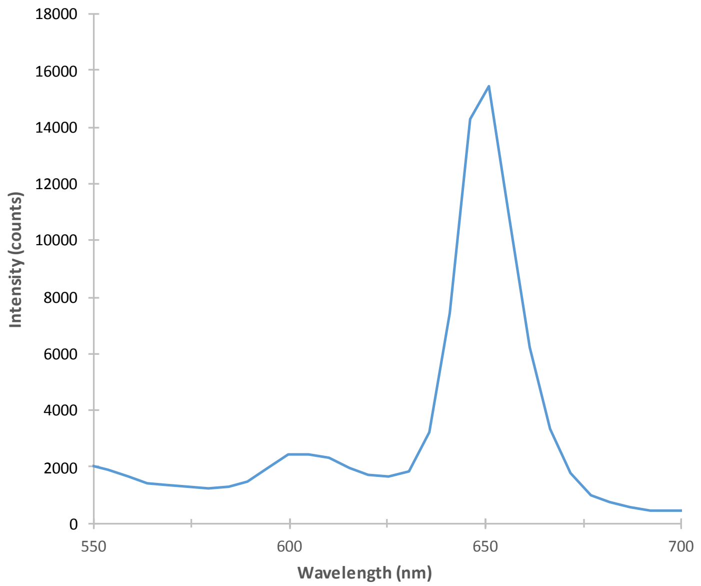

Figure S21. Emission spectrum of (4, Ph-Mn) in ethyl acetate.

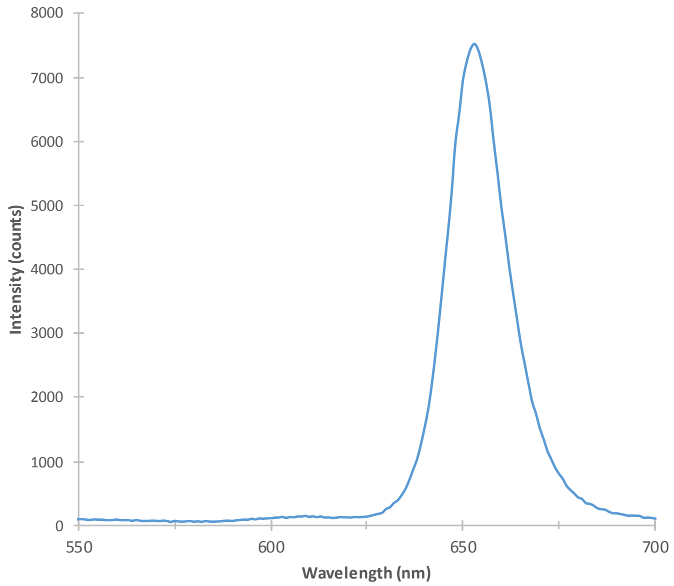

Figure S22. Emission spectrum of (5, Ph-Ni) in ethyl acetate.

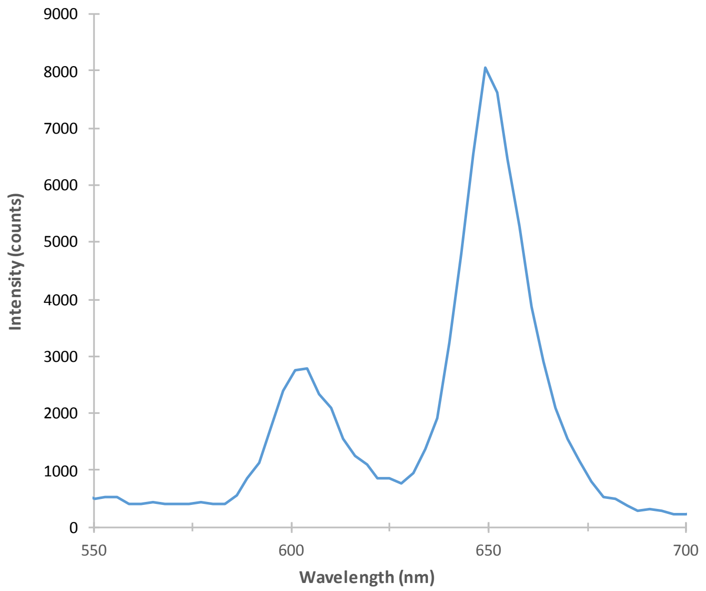

Figure S23. Emission spectrum of (6, Ph-Al) in ethyl acetate.

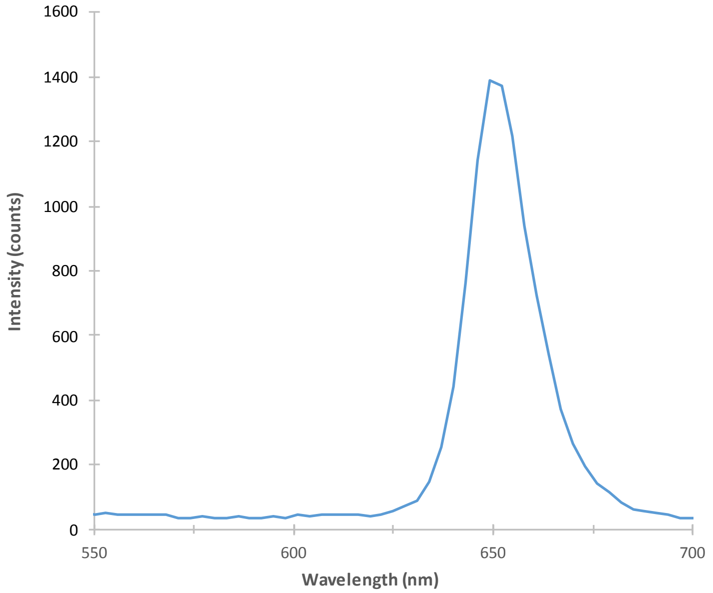

Figure S24. Emission spectrum of (7, Ph-V) in ethyl acetate.

Singlet oxygen plots

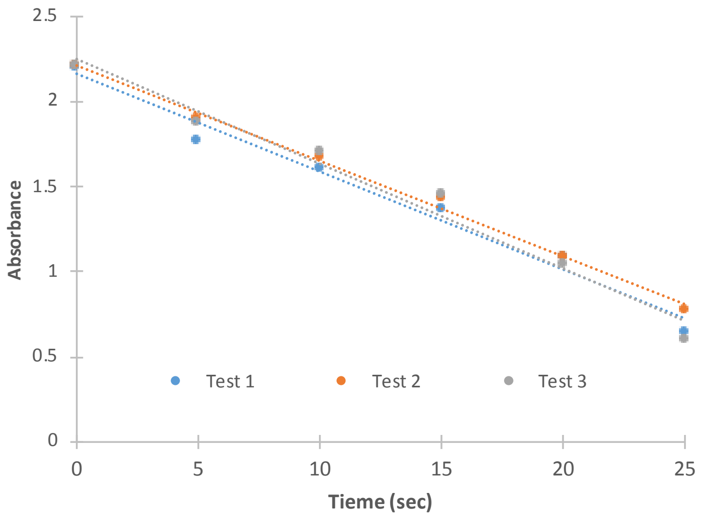

Figure S25. UV-Vis Absorbance of DPBF at 415 nm as a function of reaction time for (1, Ph), three tests are shown. Linear fitting is also shown.

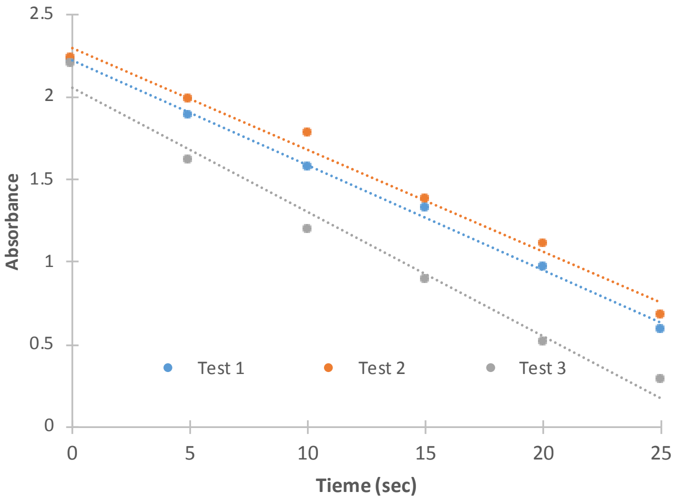

Figure S26. UV-Vis Absorbance of DPBF at 415 nm as a function of reaction time for (2, Ph-Zn), three tests are shown. Linear fitting is also shown.

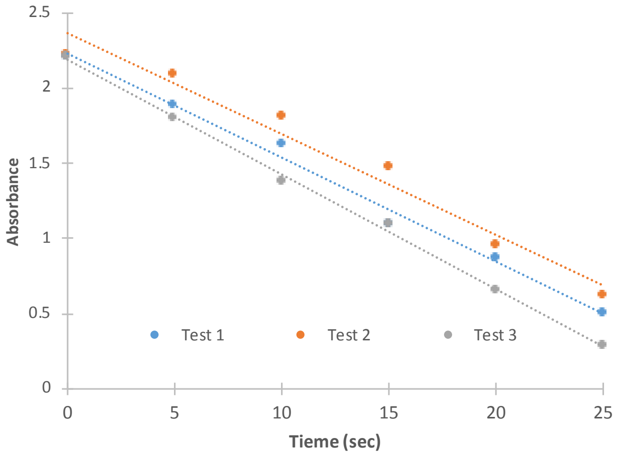

Figure S27. UV-Vis Absorbane of DPBF at 415 nm as a function of reaction time for (3, Ph-Sn), three tests are shown. Linear fitting is also shown.

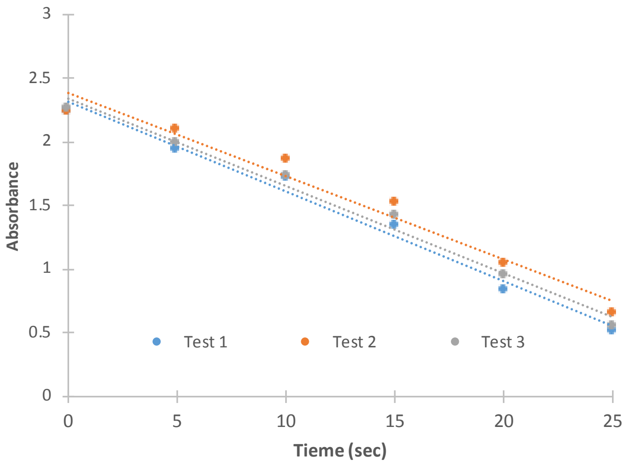

Figure S28. UV-Vis Absorbance of DPBF at 415 nm as a function of reaction time for (4, Ph-Mn), three tests are shown. Linear fitting is also shown.

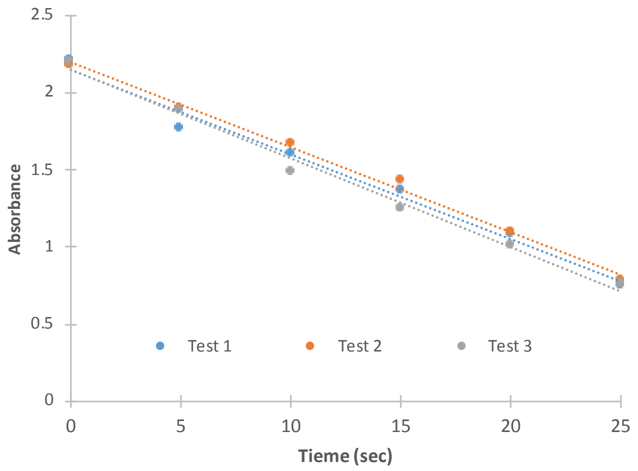

Figure S29. UV-Vis Absorbance of DPBF at 415 nm as a function of reaction time for (5, Ph-Ni), three tests are shown. Linear fitting is also shown.

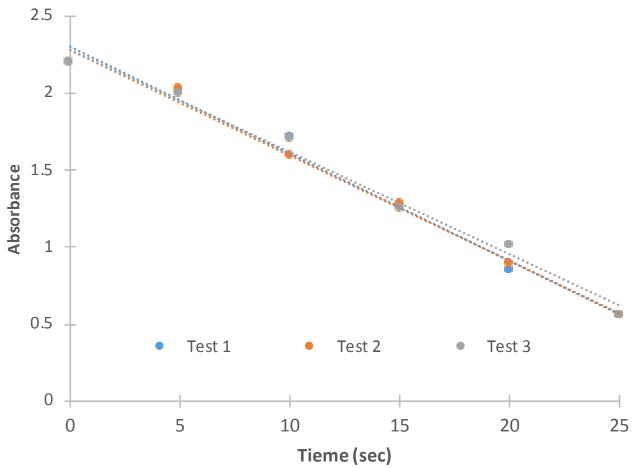

Figure S30. UV-Vis Absorbance of DPBF at 415 nm as a function of reaction time for (6, Ph-Al), three tests are shown. Linear fitting is also shown.

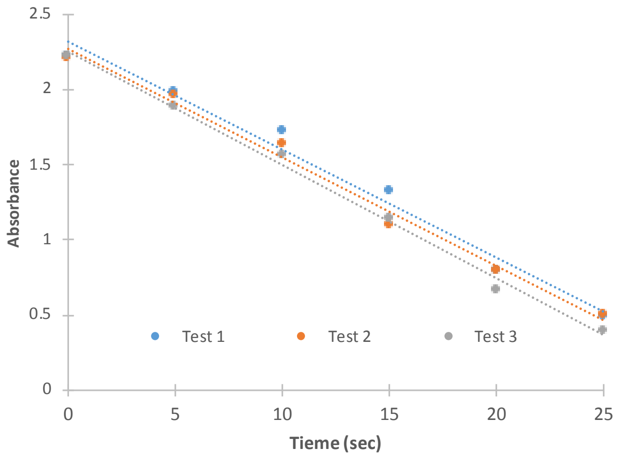

Figure S31. UV-Vis Absorbance of DPBF at 415 nm as a function of reaction time for (7, Ph-V), three tests are shown. Linear fitting is also shown.
